# Engineered Macrophage Membrane‐Coated S100A9‐siRNA for Ameliorating Myocardial Ischemia‐Reperfusion Injury

**DOI:** 10.1002/advs.202403542

**Published:** 2024-09-12

**Authors:** He Lu, Junzhuo Wang, Ziwei Chen, Jing Wang, Yaohui Jiang, Zequn Xia, Ya Hou, Pingping Shang, Rutian Li, Yuyong Liu, Jun Xie

**Affiliations:** ^1^ Nanjing Drum Tower Hospital Drum Tower Clinical College Nanjing University of Chinese Medicine No. 321 Zhongshan Road Nanjing 210008 China; ^2^ Department of Cardiology Affiliated Hospital of Nantong University Nantong 226001 China; ^3^ Nanjing Drum Tower Hospital Affiliated Hospital of Medical School Nanjing University No. 321 Zhongshan Road Nanjing 210008 China; ^4^ Department of Cardiology The People's Hospital of Jiawang District of Xuzhou Xuzhou 221011 China; ^5^ Department of Oncology Nanjing Drum Tower Hospital Affiliated Hospital of Medical School Nanjing University No. 321 Zhongshan Road Nanjing 210008 China; ^6^ Department of Cardiac Surgery National Cardiovascular Disease Regional Center for Anhui the First Affiliated Hospital of Anhui Medical University Hefei 230022 China; ^7^ Beijing Institute of Heart Lung, and Blood Vessel Diseases Beijing Anzhen Hospital Affiliated to Capital Medical University Beijing 100029 China

**Keywords:** engineered macrophage membrane, myocardial ischemia‐reperfusion injury, neutrophils, S100A8 / A9, siRNA

## Abstract

Despite the widespread adoption of emergency coronary reperfusion therapy, reperfusion‐induced myocardial injury remains a challenging issue in clinical practice. Following myocardial reperfusion, S100A8/A9 molecules are considered pivotal in initiating and regulating tissue inflammatory damage. Effectively reducing the S100A8/A9 level in ischemic myocardial tissue holds significant therapeutic value in salvaging damaged myocardium. In this study, HA (hemagglutinin)‐ and RAGE (receptor for advanced glycation end products)‐ comodified macrophage membrane‐coated siRNA nanoparticles (MMM/RNA NPs) with siRNA targeting S100A9 (S100A9‐siRNA) are successfully prepared. This nanocarrier system is able to target effectively the injured myocardium in an inflammatory environment while evading digestive damage by lysosomes. In vivo, migration of MMM/RNA NPs to myocardial injury lesions is confirmed in a myocardial ischemia‐reperfusion injury (MIRI) mouse model. Intravenous injection of MMM/RNA NPs significantly reduced S100A9 levels in serum and myocardial tissues, further decreasing myocardial infarction area and improving cardiac function. Targeted reduction of S100A8/A9 by genetically modified macrophage membrane‐coated nanoparticles may represent a new therapeutic intervention for MIRI.

## Graphical abstract

This study presents a nanocarrier system that utilizes the assembly of engineered macrophage membranes and cationic polymers to transport siRNA. This nanocarrier system can effectively target the damaged myocardium while avoiding degradation by lysosomes. Genetically modified macrophage membrane‐coated nanoparticles targeting the reduction of S100A8/A9 could serve as a novel therapeutic approach for myocardial ischemia‐reperfusion injury.

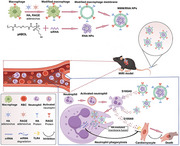

## 1 Introduction

Cardiovascular diseases, especially myocardial infarction, and their related complications remain the leading causes of death worldwide. Although reperfusion is the most logical strategy to prevent the progression of evolving myocardial necrosis, there is evidence that reperfusion through revascularization can trigger a series of events, accelerating and prolonging post‐ischemic injury. This is the concept of myocardial ischemia‐reperfusion injury (MIRI).^[^ ^1^ ^]^

The predominant pathogenic mechanism underlying MIRI is characterized by the infiltration of innate immune cells, resulting in inflammatory damage.^[^ ^2^ ^]^ S100A8/A9, consisting of the major leukocyte proteins known as the principal damage‐associated molecular patterns, recruits innate immune cells to the site of injury, thereby initiating and exacerbating tissue inflammation and myocardium injury following reperfusion. Inhibiting myocardial cell death is crucial for protecting cardiac function after reperfusion.^[^ ^3^ ^]^ S100A9 is a Ca^2+^‐binding protein that is strongly associated with pro‐inflammatory functions of neutrophils when it forms a heterodimer with its S100A8 partner.^[^ ^4^ ^]^ Deletion of S100A9 in S100A9‐knockout mice results in deletion of both S100A9 and S100A8, possibly because of the higher turnover of isolated S100A8 in the absence of its binding partner S100A9.^[^ ^5^ ^]^ S100A9 gene knockout significantly reduces myocardial cell death and improves cardiac function, and S100A9 overexpression aggravates myocardial injury.^[^ ^6^ ^]^ Therefore, exploring effective strategies to suppress the production of S100A9 during the early stages of myocardial reperfusion, thereby reducing the binding of S100A8/A9 to TLR4, is of paramount importance in decreasing myocardial inflammatory damage and preserving cardiac function.

RNA interference (RNAi) holds significant promise for diverse therapeutic strategies due to its specific gene‐silencing capability.^[^ ^7^ ^]^ Nonetheless, the clinical translation of RNAi has encountered significant obstacles primarily stemming from the limited bioavailability of small interfering RNA (siRNA).^[^ ^8^ ^]^ The limitations arise from the pronounced susceptibility of siRNA to enzymatic hydrolysis, its short half‐life, off‐target effects, suboptimal cellular uptake, and potential immunogenicity.^[^ ^9^ ^]^

Nanoparticles (NPs) are highly biocompatible and effective for diagnostics, bioimaging, and precision treatment of diseases, so the combination of NPs with siRNA is a promising direction.^[^ ^10^ ^]^ Surface characterization of NPs is vital, as NPs with inappropriate surface features are identified and eliminated by the immune system upon injection into the body.^[^ ^11^ ^]^ To overcome this obstacle, employing cell‐modified NPs can endow the nanosystem with novel characteristics, enabling diverse functionalities including immune evasion and disease targeting.

The macrophage membrane has been successfully applied in the development of biomimetic drug delivery systems due to the following characteristics^[^ ^12^ ^]^: 1) The delivery system utilizing macrophages can shield the therapeutic cargo from detection and elimination by the mononuclear phagocyte system, thereby enhancing the pharmacokinetic characteristics of the administered drugs. 2) Macrophages can utilize their pattern recognition receptors to damage molecular patterns; hence, the delivery system utilizing macrophages exhibits better inflammatory chemotaxis effects.^[^ ^13^ ^]^

However, the original therapeutic methods utilizing cell membranes have limited bioactivity and monofunctionality. Therefore, innovative methods that emphasize the enhancement of cell membrane functions before membranes are combined with biomaterials have attracted great attention in the medical field.^[^ ^14^ ^]^ In this study, we attempt to address this challenge through the overexpression of hemagglutinin (HA) and receptor for advanced glycation end products (RAGE) proteins on the membrane of macrophages. Due to the endosomal escape mediated by HA, the nanocarrier system should have higher cell penetration. The overexpression of RAGE allows the nanocarrier system to better target the highest expression site of the S100A9 protein (infarcted area), while also facilitating more efficient binding with circulating S100A9 to suppress its pro‐inflammatory effects. Meanwhile, we combined a newly generated bioreducible, linear, cationic polymer called “pABOL” to further enhance the delivery efficiency of siRNA.^[^ ^15^ ^]^

**Figure 1:**
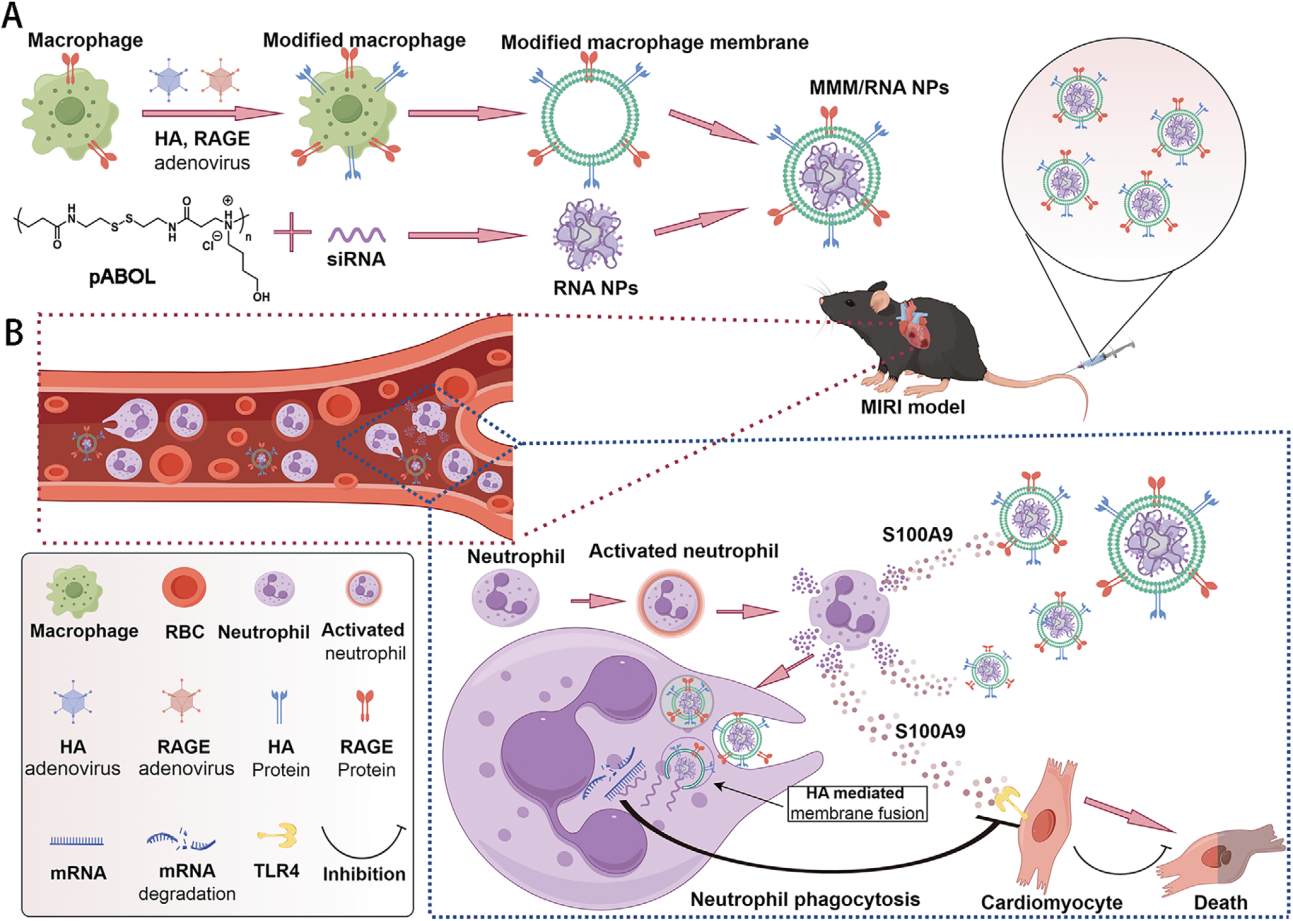
Schematic representation of MMM/RNA NPs‐mediated siRNA delivery for the treatment of MIRI. A) Depiction of the MMM/RNA NPs preparation. Macrophages were transfected with adenoviruses encoding for HA and RAGE to construct engineered macrophages. Engineered macrophage membranes were isolated and coated onto pABOL nanoparticle cores loaded with siRNA to prepare MMM/RNA NPs. B) Diagram of MMM/RNA NPs injected into the tail vein of myocardial ischemia‐reperfusion mice. Myocardial ischemic injury recruits a large number of neutrophils in the blood, which activate and release S100A9 inflammatory factors. MMM/RNA NPs rely on their cell membrane protein RAGE to recruit to the myocardial injury area along the concentration of S100A9. Neutrophils engulf MMM/RNA NPs, and MMM/RNA NPs rely on their cell membrane protein HA to play an endosomal escape role, transporting siRNA to the cytoplasm for silencing. Further, siRNA recognizes and degrades mRNA, reduces the level of S100A9, inhibits the binding of S100A9 to TLR4, and thus reduces mitochondrial dysfunction in cardiomyocytes.

The combination of engineered macrophage membranes and the pABOL cationic polymer holds the potential to create a highly efficient, targeted, and safe siRNA delivery system. In this study, we successfully prepared modified macrophage membrane‐coated siRNA NPs (MMM/RNA NPs) using the cell membrane and the pABOL cationic polymer as carriers for siRNA targeting S100A9 (S100A9‐siRNA). The MMM/RNA NPs exhibited outstanding S100A9 silencing efficacy and demonstrated a robust capability to ameliorate myocardial infarction, owing to their superior biocompatibility (**Figure** **1**).

## 2 Results and Discussion

### 2.1 Modification of Macrophages and Characterization of MMM/RNA NPs

Macrophage membranes were selected as the optimal choice for membrane coating due to the abundance of functional proteins that facilitate inflammatory responses and cellular recruitment during MIRI.^[^ ^16^ ^]^ RAGE is specifically expressed on macrophages and exhibits the ability to interact with S100A9, thereby facilitating the recruitment of cells to the site of myocardial ischemic injury.^[^ ^6b^ ^]^ In the natural setting, the influenza virus utilizes its surface HA protein to facilitate the transport of its genetic material into the cytosol, enabling its evasion from the intracellular compartment and mitigating potential harm.^[^ ^17^ ^]^ To replicate this mechanism, we engineered biomimetic nanoparticles by coating them with a macrophage membrane enriched with HA on their surface. These NPs (MMM/RNA NPs) demonstrated the capacity to evade endosomes and efficiently release siRNA into the cytosol in the following experimental studies.

**Figure 2:**
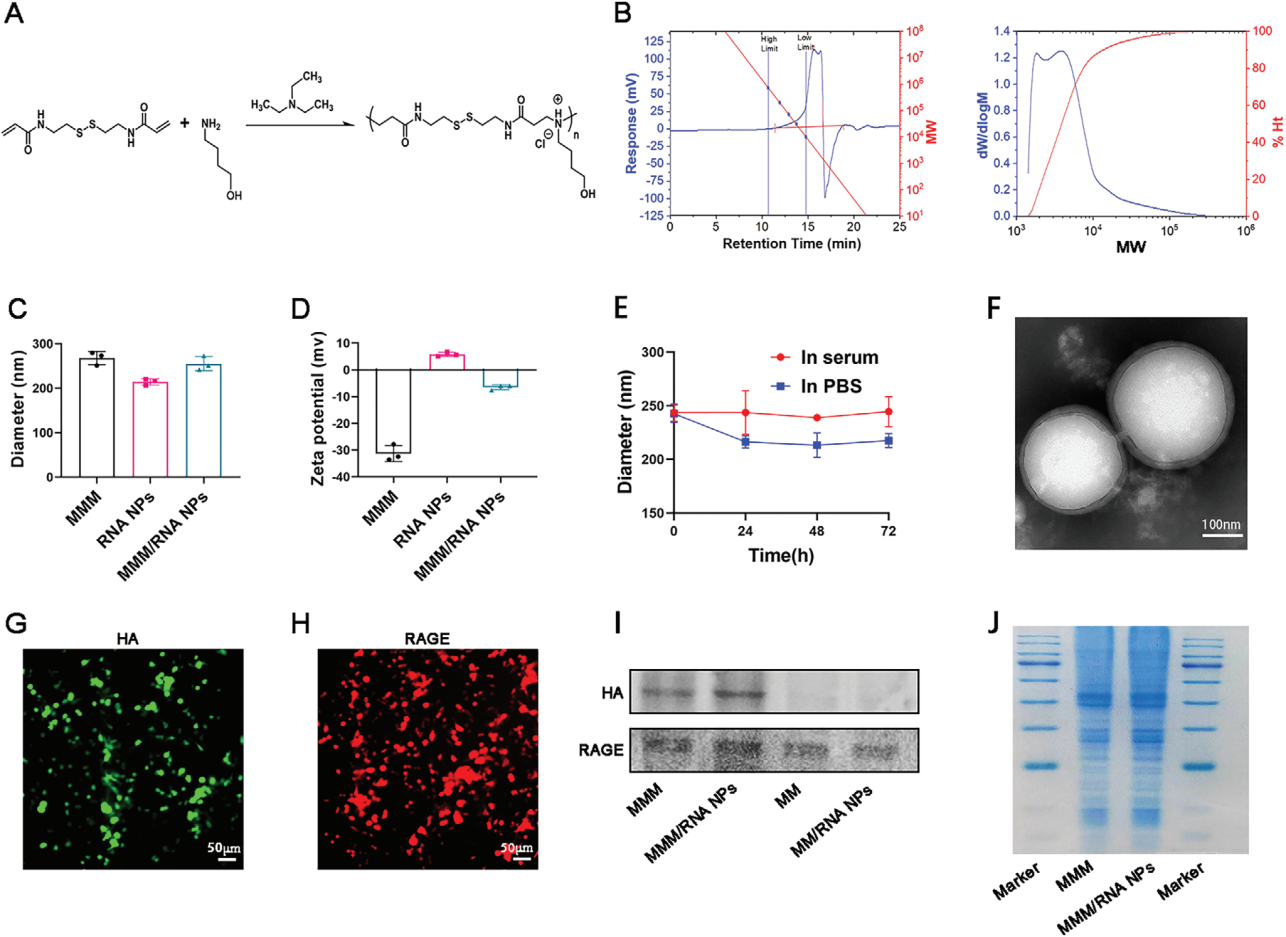
Synthesis of pABOL and characterization of nanoparticles. A) Schematic illustration of improved aza‐Michael addition to synthesize pABOL. Polymerization reaction of ABOL with CBA in the presence of triethylamine (TEA). B) The molecular weight of pABOL measured by GPC. C,D) Size (C) and surface zeta potential (D) of MMM, RNA NPs, and MMM/RNA NPs as measured by dynamic light scattering. E) Stability of MMM/RNA NPs in 1× PBS or in serum, determined by monitoring particle size (diameter, nanometers), over a 72 h period. F) TEM images of MMM/RNA NPs (scale bar = 100 nm). G, H) CLSM images of RAW264.7 cells after transfection with adenovirus encoding for H7 and RAGE for 48 h. I) Representative protein bands of MM, RNA NPs, MMM, and MMM/RNA NPs measured by western blot. J) Total protein content visualization of modified macrophage membrane‐derived vesicles (MMM) and modified macrophage membrane‐coated nanoparticles (MMM/RNA NPs) stained with Coomassie Brilliant Blue.

To prepare siRNA‐loaded pABOL NPs (RNA NPs), we initially synthesized pABOL through the aza‐Michael polyaddition reaction between 4‐amino‐1‐butanol (ABOL) and N, N'‐cysteamine bisacrylamide (CBA) in the presence of triethylamine (TEA) (**Figure** **2**A). Previous studies have demonstrated that pABOL with a molecular weight of 8 kDa exhibits high transfection efficiency and low cytotoxicity.^[^ ^15^ ^]^ In our study, we synthesized pABOLs with a molecular weight of 8.1 kDa (Figure 2B). The pABOL stock solution was prepared by dissolving pABOL directly in molecular grade water, which was further mixed with siRNA to obtain RNA NPs. Subsequently, the RNA NPs were coated with modified macrophage membranes (MMMs) using an extrusion method.^[^ ^18^ ^]^ The membranes were derived from modified macrophages that overexpressed HA tagged with green fluorescent protein (HA‐GFP) and RAGE tagged with mCherry (RAGE‐mCherry) on their surfaces, and these were used for coating the RNA NPs. Dynamic light scattering (DLS) analysis revealed that upon membrane encapsulation, the size of MMM/RNA NPs increased by ≈20 nm compared to the size of RNA NPs, corresponding to the thickness of the coated macrophage membrane (Figure 2C). Furthermore, MMM/RNA NPs exhibited a reduced negative zeta potential compared to the neutral surface charge of RNA NPs, a characteristic known to confer prolonged blood circulation compared to NPs with neutral or positive surface charges (Figure 2D).^[^ ^19^ ^]^ The stability of MMM/RNA NPs related to size and membrane coating was assessed over a 96 h period in phosphate buffered saline (PBS) and 10% fetal bovine serum (FBS), demonstrating excellent preservation as confirmed by DLS measurements (Figure 2E).^[^ ^20^ ^]^ Transmission electron microscopy analysis of negatively stained MMM/RNA NPs revealed a core‐shell structure with a spherical shape, where the membrane was accurately coated onto the polymeric cores (Figure 2F).^[^ ^21^ ^]^ Given that the RNA NPs were coated with membranes that had enhanced HA and RAGE expression, the expression of these two proteins on MMM/RNA NPs was characterized using confocal laser scanning microscopy (CLSM) (Figure 2G,H) and western blot analysis (Figure 2I). In addition, to determine whether proteins on modified macrophage membranes had been successfully translocated to the surface of RNA NPs, protein analysis was performed using Coomassie blue staining.^[^ ^22^ ^]^ MMM/RNA NPs and modified macrophage membranes had similar total protein profiles, indicating that most of the proteins of the modified macrophage membrane were translocated to the surface of the RNA NPs, as shown in Figure 2J.

### 2.2 Endosomal Escape and Targeted Binding of S100A9 In Vitro

**Figure 3:**
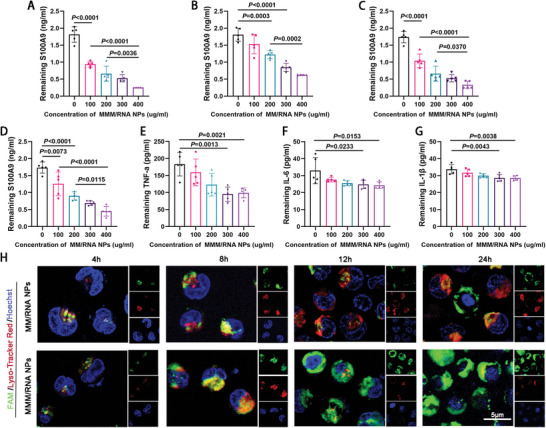
Endosomal escape in vitro. A,B) Binding capacity of MMM/RNA NPs and MM/RNA NPs with directly purchased S100A9 after incubated for 1 h examined by ELISA (n = 5). C,D) Binding capacity of MMM/RNA NPs and MM/RNA NPs with S100A9 produced by neutrophils after incubated for 24 h examined by ELISA. E–G) Binding capacity of MMM/RNA NPs with TNF‐a, IL‐6 and IL‐1β produced by neutrophils after incubated for 24 h examined by ELISA. H) Fluorescent visualization of MNHCs incubated with MM/RNA NPs and MMM/RNA NPs for 4, 8, 12 and 24 h (green: nanoparticles, red: endosomes, blue: nuclei). scale bar = 5 µm. Results are expressed as mean ± SD. One‐way analysis of variance (ANOVA) was used for the analysis in A–H).

During inflammation, macrophage membranes play a crucial role in the absorption and neutralization of S100A9 through the interaction with RAGE.^[^ ^23^ ^]^ In our study, we camouflaged the MMM/RNA NPs with engineered macrophage membranes that exhibited enhanced expression of RAGE. To evaluate the binding capacity of MMM/RNA NPs to S100A9, we conducted an incubation experiment with varying concentrations of MMM/RNA NPs or MM/RNA NPs.^[^ ^24^ ^]^ The MMM/RNA NPs/S100A9 mixture were incubated at 37 °C for 1 h. After removing the NPs, the remaining cytokines in the supernatant were quantified. The results in **Figure** **3**A demonstrate a concentration‐dependent decrease in the residual level of S100A9 in the supernatant of the MMM/RNA NPs/S100A9 mixture, indicating the binding of MMM/RNA NPs to S100A9. Similarly, in the supernatant of the MM/RNA NPs/S100A9 mixture, a decrease in the residual level of S100A9 was observed (Figure 3B), suggesting that MMM/RNA NPs exhibited a stronger binding capacity to S100A9 than MM/RNA NPs.

To further assess the adsorption capacity of polymer NPs, an alternative method was employed. Mouse neutrophil cells (MNHCs) were stimulated with 100 ng mL^−1^ LPS, and the secretion of cytokine was quantified. MNHCs were then treated with MMM/RNA NPs or MM/RNA NPs at various concentrations for 24 h. Gradual reduction in the level of S100A9 in the supernatant was observed as the concentration of MMM/RNA NPs and MM/RNA NPs increased. Remarkably, the attenuation of S100A9 levels was more pronounced in the MMM/RNA NP treatment group (Figure 3C,D). Additionally, a modest decrease in the levels of tumor necrosis factor alpha (TNF‐α), interleukin‐6 (IL‐6), and interleukin‐1β (IL‐1β) in the supernatant compared to S100A9 was observed, suggesting the presence of receptor proteins for TNF‐α, IL‐6, and IL‐1β on the surface of the MMM (Figure 3E–G).

A qualitative approach was utilized to assess the intracellular escape and avoidance of degradation of MMM/RNA NPs in vitro.^[^ ^25^ ^]^ MNHCs were exposed to MM/RNA NPs or MMM/RNA NPs containing carboxyfluorescein‐labeled (FAM‐labeled) siRNAs for 4, 8, 12, and 24 h, and subsequently stained with Lyso‐Tracker Red (Lysosomal red fluorescent probe) and Hoechst 33 342 (Blue fluorescent dye for cell nucleus) before imaging (Figure 3H). Cellular uptake of MM/RNA NPs and MMM/RNA NPs was observed using CLSM. At the 4 h timepoint, colocalization of NPs (FAM‐labeled) and endosomes (Lyso‐Tracker Red labeled) was observed, indicating endocytosis for both formulations. At the 12 h timepoint, external NPs signals were detected for MMM/RNA NPs, indicating successful endosomal escape, while the intracellular signal of MM/RNA NPs still colocalized with the endosomes. After 24 h of incubation, signals from MMM/RNA NPs had penetrated into the cytoplasm, whereas minimal evidence of cytoplasmic delivery of MM/RNA NPs was observed. Importantly, cells treated with MMM/RNA NPs exhibited a reduced Lyso‐Tracker Red signal, indicating a decrease in lysosomal load due to successful lysosomal escape.^[^ ^26^ ^]^

### 2.3 In Vitro Safety Assessment

To evaluate the biological effects, NPs, MM/RNA NPs, and MMM/RNA NPs were incubated with MNHCs, HUVECs, SMCs, and RAW 264.7 cells, respectively. The cytotoxicity of these NPs was assessed using the Cell Count Kit 8 (CCK‐8) method. The results indicated no significant difference between the treatment groups and the untreated group, demonstrating favorable cell compatibility of our drug delivery system (Figure S1, Supporting Information).

Blood compatibility is a critical criterion for evaluating the biocompatibility of biomaterials. In vitro direct contact was employed to assess NPs‐induced hemolysis. As shown in Figure S2 (Supporting Information), no anomalies or particulate precipitation were observed in the supernatant after incubating NPs, MM/RNA NPs, and MMM/RNA NPs with blood, indicating the absence of erythrocyte damage.

### 2.4 Therapeutic Effect In Vitro

**Figure 4:**
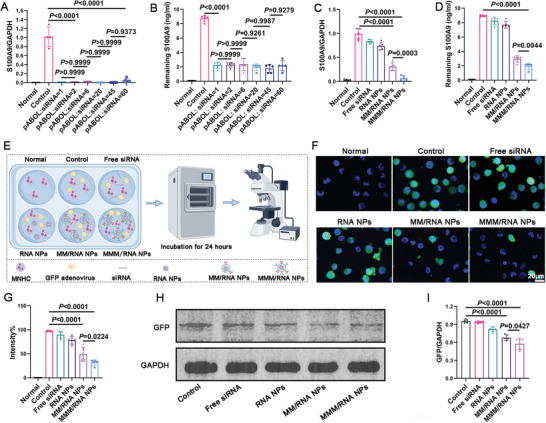
siRNA transfection in vitro. A, B) Inhibition effect of pABOL to siRNA at different pABOL/siRNA weight ratios, ranging from 1 to 60, quantified by real‐time quantitative polymerase chain reaction (qPCR) (A) and ELISA (B) (n = 5). C, D) S100A9 levels detected by qPCR and ELISA after treatment in different treatment groups. E) Schematic diagram of co‐incubation of MNHCs, adenovirus, and nano‐carrier system to detect the effect of nano‐carrier system delivery. F,G) Fluorescence images and quantification of different treatment groups after 24 h of co‐incubation (blue: nuclei) (n = 5). scale bar = 20 µm. H,I) Western blot and quantification of GFP expression levels. Results are presented as mean ± SD. One‐way ANOVA was used for the analysis in A‐D,G,I).

Subsequently, we assessed the efficacy of MMM/RNA NPs in facilitating siRNA‐mediated knockdown of the target gene in vitro. As previously demonstrated, MMM/RNA NPs exhibited negligible cytotoxicity across all cell lines at concentrations ranging from 10 to 200 µg mL^−1^. Within this concentration range, optimal inhibition of target gene expression by MMM/RNA NPs was observed at a pABOL/siRNA ratio of 2:1 (**Figure** **4**A–D). MNHCs were co‐incubated with adenovirus transfected with green fluorescent protein (GFP) gene, followed by the addition of PBS, free siRNA, RNA NPs, MM/RNA NPs, and MMM/RNA NPs containing GFP siRNA. Next, we examined the inhibitory effect of GFP siRNA transported by different transport systems (PBS, free siRNA, RNA NPs, MM/RNA NPs, and MMM/RNA NPs) by confocal microscopy to react to the transport effect of different transport systems, thereby demonstrating the therapeutic effect. After 24 h, fluorescence microscopy images clearly depicted a reduction in green fluorescence, indicative of decreased GFP gene expression in MNHCs upon treatment with MMM/RNA NPs (Figure 4E–G). The corresponding protein bands also demonstrated significant GFP protein inhibition in the MMM/RNA NPs treatment group (Figure 4H,I).

### 2.5 Targeting Research In Vivo

**Figure 5:**
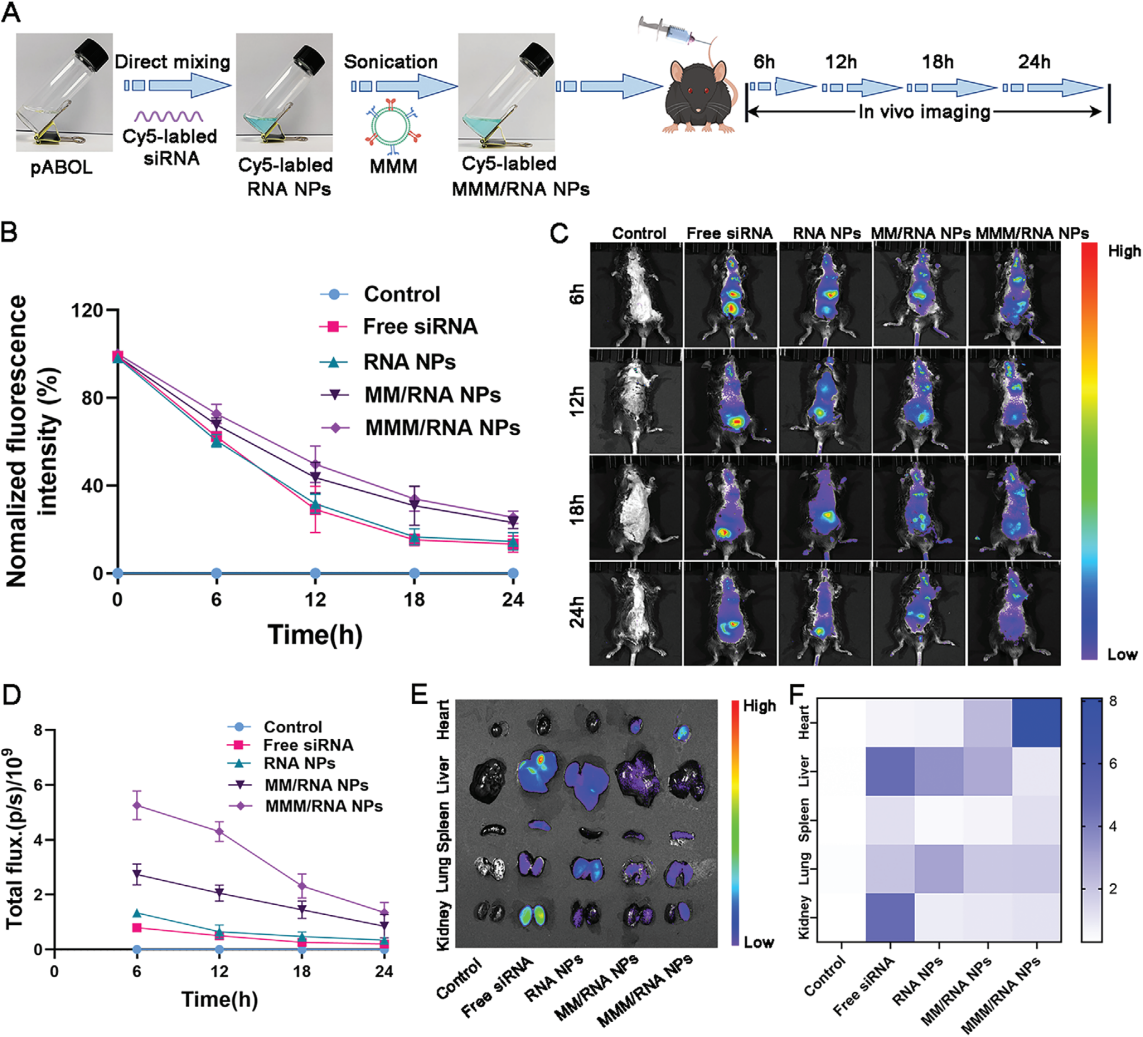
In vivo targeting capacity of MMM/RNA NPs in mouse model of MIRI. A) Synthesis process of MMM/RNA NPs containing Cy5‐labeled siRNAs. B) Circulating profiles of PBS, free siRNA, RNA NPs, MM/RNA NPs, and MMM/RNA NPs after intravenous injection in mice. C) Representative in vivo images of MIRI mice at different time intervals after intravenous injection of PBS, free siRNA, RNA NPs, MM/RNA NPs, and MMM/RNA NPs (n = 3). D) Quantification of fluorescence intensity in hearts at different time intervals after intravenous injection of PBS, free siRNA, RNA NPs, MM/RNA NPs and MMM/RNA NPs. E) Representative ex vivo fluorescence images of Cy5 fluorescent dye accumulation in different organs 6 h after intravenous injection of Cy5‐labelled nanoparticles. F) Quantification of fluorescence intensity of major organs. Results are presented as mean ± SD.

Before investigating the in vivo targeting of MMM/RNA NPs, we prepared Cy5‐labeled NPs (**Figure** **5**A). Circulation time is critical for NPs to exert targeting effects, and prolonged circulation time promotes the recruitment of more NPs to the target organ.^[^ ^27^ ^]^ To assess the cycle time of the NPs, the intensity of the fluorescently labeled NPs was evaluated by calculating the fluorescence intensity of the NPs at different time points. As shown in Figure 5B, the fluorescence intensity of MMM/RNA NPs at different times was higher than that of the other groups, suggesting that MMM/RNA NPs have a longer cycling time due to the role played by the coated engineered macrophage membranes.

We investigated the targeted delivery effect of MMM/RNA NPs on damaged myocardium in a mouse model of myocardial ischemia‐reperfusion (MIRI). Equal quantities of PBS, free siRNA, RNA NPs, MM/RNA NPs, and MMM/RNA NPs with Cy5 at an injection dose of 2.0 mg kg^−1^ were administered to the MIRI mice. Fluorescence intensity in the major organs of the mouse (heart, liver, spleen, lungs, and kidneys) was visualized and quantified at set time points using the in vivo imaging system (IVIS).^[^ ^28^ ^]^ Injections of MMM/RNA NPs and MM/RNA NPs accumulated significantly more in the heart compared to the control, free siRNA, and RNA NPs‐treated groups, facilitated by macrophage chemotactic targeting. Furthermore, MMM/RNA NPs exhibited stronger fluorescence signals in the heart than the MM/RNA NPs‐treated group, indicating superior targeting to ischemically injured myocardium. The fluorescent signal of siRNA was also detected in other major organs, predominantly in the liver and kidneys, likely attributed to the reticuloendothelial system clearance in the liver and kidneys following direct injection of NPs into the bloodstream (Figure 5C–F).^[^ ^29^ ^]^ Overall, the experimental data suggest that MMM/RNA NPs have a longer circulation time and a higher accumulation rate in the injured heart, which facilitates cardiac repair.

### 2.6 In Vivo Therapeutic Effect

**Figure 6:**
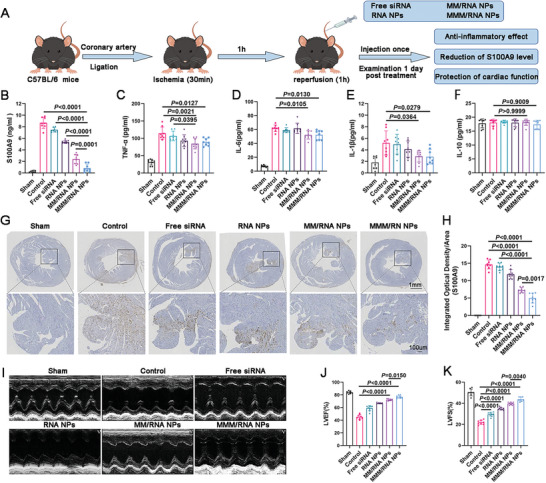
MMM/RNA NPs reduce S100A9 level and improve cardiac function. A) Schematic diagram of experimental plan. A mouse model of myocardial ischemia‐reperfusion was established by ligating the proximal left coronary artery of male C57BL/6 mice for 30 min and releasing it. B–F) The concentrations of S100A9, TNF‐α, IL‐6, IL‐1β, and IL‐10 in serum were detected by ELISA. G,H) Representative immunohistochemical staining results showing that S100A9 expression in mice heart tissues 24 h after MI/R surgery (n = 8). Scale bar: 1 mm (top) and 100 µm (bottom). I) Representative M‐mode images at 14 d post‐MI. J,K) Quantification of LVEF% and LVFS% (n = 8). Results are presented as mean ± SD. One‐way ANOVA was used for the analysis in B‐F, H, J,K).

Having observed the capacity of MMM/RNA NPs to sequester S100A9 and reduce target gene expression in cell studies, we proceeded to investigate whether the MMM/RNA NPs drug delivery system could exert anti‐inflammatory and cardioprotective effects using anti‐S100A9 siRNA in an animal model. To assess the therapeutic effects of different treatment groups (PBS, free siRNA, RNA NPs, MM/RNA NPs, MMM/RNA NPs) on MIRI, a dosage of 2.0 mg kg^−1^ of PBS (control group), free siRNA, RNA NPs, MM/RNA NPs, and MMM/RNA NPs was administered via the tail vein after 1 h of myocardial reperfusion in mice (**Figure** **6**A).

In comparison to sham‐operated mice, serum S100A9 levels were significantly elevated in MI mice (Figure 6B). Administration of free siRNA did not significantly impact serum S100A9 levels. Treatment with MM/RNA NPs and MMM/RNA NPs significantly attenuated the level of S100A9 in serum, with a greater decrease in the MMM/RNA NPs treatment group, indicating a significant anti‐inflammatory effect. Furthermore, serum levels of TNF‐α, IL‐6, and IL‐1β were examined across the different treatment groups (Figure 6C–E). Similar results were observed in serum TNF‐α, IL‐6, and IL‐1β levels following treatment with MMM/RNA NPs or MM/RNA NPs, which is consistent with the previously observed targeted binding capacity of cell membrane‐coated NPs. No significant differences were detected between the treatment groups and the control group in serum IL‐10 (Figure 6F). We also performed immunohistochemical staining of the myocardium of MIRI mice in different treatment groups, and the results showed that S100A9 levels were significantly decreased in the MM/RNA NPs and MMM/RNA NPs treatment groups, with a greater decrease in the MMM/RNA NPs treatment group (Figure 6G,H). Two weeks post‐injection, the cardioprotective effects were assessed. The left ventricular ejection fraction (LVEF%) was significantly reduced in MIRI mice, whereas it was significantly higher following treatment with MMM/RNA NPs compared to free siRNA, RNA NPs, or MM/RNA NPs (Figure 6I,J). Additionally, MMM/RNA NPs treatment significantly improved left ventricular fractional shortening (LVFS%) (Figure 6K).

### 2.7 MMM/RNA NPs Reduced Mortality In Mice

**Figure 7:**
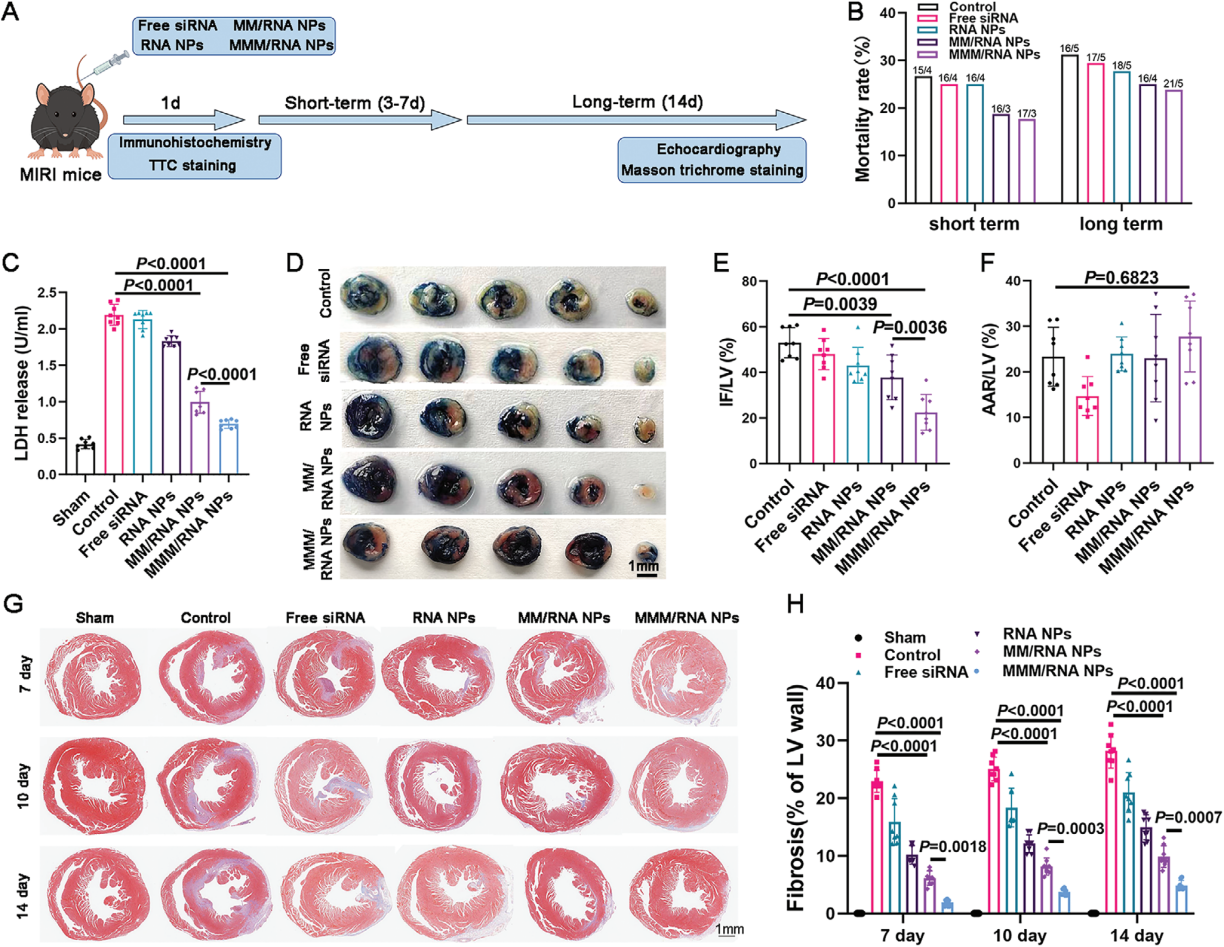
MMM/RNA NPs attenuate myocardial injury and improve survival. A) Schematic diagram of the experimental program. B) Mortality rate in different treatment groups. C) Serum levels of myocardial cell death marker lactate dehydrogenase (LDH) 24 h after MIRI. D‐F) Triphenyltetrazolium chloride (TTC) staining of heart sections collected from Control, free siRNA, RNA NPs, MM/RNA NPs, and MMM/RNA NPs (n = 8 mice each). Hearts were collected 24 h after reperfusion to assess infarct area (IF), area at risk (AAR), and left ventricle (LV). Scale bar: 1 mm. G) Representative Masson trichrome staining of infarcted hearts (blue, scar tissue; red, surviving myocardium) after 2 weeks of treatment. Scale bar:1 mm. H) Area of fibrosis in the left ventricular (LV) wall at different time points. Results are presented as mean ± SD. One‐way ANOVA was used for the analysis in C,E,F,H).

To better assess the therapeutic effects of MMM/RNA NPs, we monitored the mortality of mice in different treatment groups. The mortality rate in the post‐MIRI treatment groups (MMM/RNA NPs and MM/RNA NPs groups) was lower than that in the control (PBS), free siRNA, and RNA NPs groups, suggesting that biomimetic macrophage membrane coating reduces the mortality rate in both the long‐term (14 days) and the short‐term (3‐7 days) groups (**Figure** **7**A,B). Mice treated with MMM/RNA NPs had an even lower mortality rate, suggesting that engineered macrophage membrane coatings on siRNA nanoparticles exhibited better targeting capabilities for improved therapeutic outcomes. Previous findings suggest that S100A8/A9 is critical for I/R‐induced myocardial death and heart failure.^[^ ^30^ ^]^ Studies in the previous section have shown that MMM/RNA NPs have a significant reduction in S100A9, and consistent with this, myocardial death was inhibited in the MMM/RNA NPs‐treated group after MIRI, as evidenced by a reduction in lactate dehydrogenase (LDH) (Figure 7C) and cardiac troponin I (CTnI) in serum (Figure S3A, Supporting Information). We next examined the levels of ANP and BNP in serum of MIRI mice and found similar results, with the MM/RNA NPs and MMM/RNA NPs treatments having significantly lower levels of ANP and BNP than the other treatment groups, with the MMM/RNA NPs treatment having the lowest levels of ANP and BNP (Figure S3B,C, Supporting Information). Triphenyltetrazolium chloride (TTC) staining showed a reduction in the size of the infarcted area after treatment with MM/RNA NPs, but the infarcted area was more significantly reduced after 24 h in the MMM/RNA NPs‐treated group (Figure 7D–F). After injection for 7, 10, and 14 days, Masson's trichrome staining of heart cross‐sections revealed distinct areas of myocardial fibrosis in control MIRI mice (Figure 7G). MIRI mice treated with MMM/RNA NPs and MM/RNA NPs exhibited a significant reduction in the area of myocardial fibrosis compared to the control group (Figure 7H).

### 2.8 Biosafety Assignment

**Figure 8:**
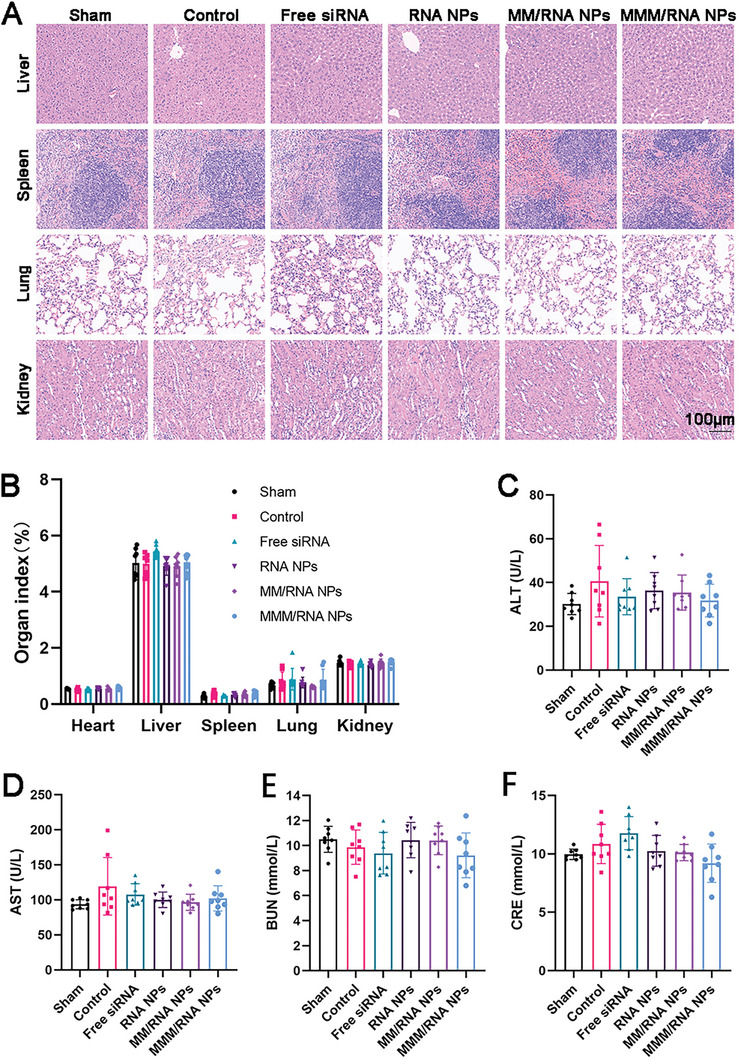
Biosafety of MMM/RNA NPs in vivo. A) HE staining of tissue sections of major organs 14 d after injection. Scale bar: 100 µm. B) Organ body weight ratio of the myocardial ischemia‐reperfusion injury mice in different treatment groups (mean ± SD, n = 8). C–F) Biochemical markers relevant to hepatic and kidney function (mean ± SD, n = 8 independent experiments).

No significant tissue organ HE staining changes were observed between PBS, free siRNA, RNA NPs, MM/RNA NPs, and MMM/RNA NPs administrations, suggesting that the NPs require minor safety concerns (**Figure** **8**A). To further assess the potential occurrence of adverse events such as edema or organ injury (heart, liver, spleen, lung, kidney) in each treatment group, the organ‐to‐body weight ratio was measured. The experimental findings revealed no significant differences between the treatment and control groups (Figure 8B), indicating that the administration of different nanoparticles did not have a significant adverse impact on the main organs. Furthermore, clinical biochemical analysis indicated normal levels of alanine aminotransferase (ALT), aspartate aminotransferase (AST), blood urea nitrogen (BUN), and creatinine (CRE), suggesting that liver and kidney function remained unaffected by the treatment (Figure 8C–F).

## 3 Conclusion

In this study, we presented a novel delivery system for engineering modified cell membrane‐coated NPs to target siRNA transport to myocardial ischemic injury lesions. The modification of HA and RAGE enhances the immunocompatibility and targeting ability of NPs. We also demonstrated that MMM/RNA NPs could effectively target binding to S100A9 and enable siRNA to evade lysosomal digestion and efficiently transport siRNA to the cytoplasm to exert silencing effects, leading to improved cardiac function and reduced mortality in mice after MIRI. Encouraged by these advanced features and results, we believe that MMM/RNA NPs may be able to address almost all extracellular and intracellular barriers associated with local siRNA delivery, and may have significant application potential in RNAi‐based disease treatment.

## 4 Experimental Section

### Materials and Cells

siRNA targeting S100A9 mRNA (S100A9‐siRNA, sense: 5′‐CCTCATAAATGACATCATG‐3′), scrambled siRNA (siN.C, sense: 5′‐GGCUCUAGAAAAGCCUAUGCdTdT −3′), FAM and Cy5‐labeled siRNA were synthesized by Guangzhou RiboBio Life Science Co. The adenovirus for mouse HA and RAGE were purchased from GENECHEM Incorporation (China). Anti‐rabbit S100A9 (D3U8M) antibody was purchased from Cell Signaling Technology (MA, USA). Recombinant mouse S100A8/A9 (8916‐S8‐050) was purchased from R&D Systems (R & D SYSTEMS, Minneapolis, USA). Mouse S100A9, mouse IL‐6, mouse IL‐1β, mouse IL‐10, mouse TNF‐α, mouse ANP, mouse BNP, and mouse CTnI ELISA kits were purchased from ELK Biotechnology (Wuhan, China). Mouse MPO‐DNA ELISA kit was purchased from Shanghai Hengyuan Biological Technology Co., LTD. ROS Assay Kit was purchased from Best Biotechnology (Shanghai, China). SOD and MDA Assay Kit was purchased from Beyotime Biotech (Shanghai, China). LDH Assay Kit was purchased from Nanjing Jiancheng Bioengineering Institute.

Murine RAW 264.7, SMC, and HUVEC lines were purchased from Nanjing BioChannel Biotechnology Co., Ltd (Nanjing, China). MNHCs were acquired from Qingqi Biotechnology Development Co., Ltd (Shanghai, China). MAEC cells were purchased from BNCC (Beijing, China). Dulbecco's Modified Eagle's medium (DMEM) was purchased from KeyGEN Biotech (China). Enhanced Cell Counting Kit‐8 was purchased from Beyotime Biotech (Shanghai, China). The bicinchoninic acid (BCA) Protein Assay kits were purchased from Absin (China).

### Modification of Macrophages

The lentiviral transfection method was used to upregulate specific proteins on the surface of RAW 264.7 cells. Preparation of HA and RAGE viral vectors was accomplished with the assistance of Genechem Co., LTD (Shanghai, China). Gene sequences for HA and RAGE can be found in Table S1 and Table S2 (Supporting Information). CLSM and western blots were used to detect the protein expression levels of HA and RAGE after modification.

### Extraction of Engineered Macrophage Membrane

To obtain bioactive membranes, macrophages were lysed using a low osmotic lysis method. The lysis solution consisted of 1 mM KCl, 1.5 mM MgCl2, and 1 mM phenylmethanesulfonylfluoride were added to 10 mM Tris‐HCl, pH = 8.0. The modified macrophage suspension was collected from six 100‐mm culture dishes and centrifuged at 900 rpm for 5 min at 4 °C. Macrophages were resuspended in precooled PBS and underwent three rounds of centrifugation. Cells were resuspended by adding 2 mL of cold lysis buffer, followed by a 15 min incubation in an ice bath. The cell suspension was then freeze‐thawed four to five times under liquid nitrogen and room temperature conditions to completely break up the cells. The suspension was centrifuged at 900 × *g* for 5 min at 4 °C and the supernatant was collected and centrifuged at 15000 × *g* for 20 min. Sediment (membrane fragments) was collected. Membrane fragments wereresuspended with an appropriate amount of lysis solution, and then the protein concentration was determined using a BCA Protein Assay Kit.

### Preparation of pABOL

pABOL was prepared using the nanoprecipitation method as described previously.^[^ ^13^ ^]^ Briefly, N, N'‐cystaminebis (acrylamide) (221.0 mg, 0.848 mmol), 4‐amino‐1‐butanol (78 µL, 0.840 mmol), and triethylamine (12 µL, 0.084 mmol) were added to a stirring bottle with a rotor and placed on a magnetic stirrer. A mixed solvent, MeOH/water (176 µL, 4/1, v/v), was also added into the ampule flask. The polymerization reaction took place in the dark at 45 °C under a static nitrogen atmosphere. The mixture was allowed to react for 32 h to produce a highly viscous solution. Once the target molecular weight was reached, the reaction was stopped by MeOH dilution (50 mL). The reaction mixture was then acidified with 1.0 M HCl to pH∼4 and then purified by dialysis (molecular weight cutoff = 3.5 kDa) with acidic water (4.0 L, pH∼5, refreshed 6 times in 3 d). After freeze‐drying, the polymers in the form of HCl‐salt were collected as a white solid.

### Preparation of RNA NPs

The reserve solution of pABOL was prepared by directly dissolving white solid form of pABOL in ultrapure water (DNase/RNase‐Free, sterile), which and can be stored at 4 °C for up to 3 months. First, 5 µl of siRNA reserve solution was added to 250 µl of HEPES buffer (20 mM HEPES, 5 wt% glucose aqueous solution, pH 7.4). An aliquote of 10 µl pABOL reserve solution was diluted to 510 µL using the same buffer. Each tube was vortexed to ensure uniformity. Then, the pABOL buffer solution was added to the siRNA buffer solution rapidly and vortexed for 20 sec to form a mixture.

### Preparation of Cell‐Membrane‐Coated siRNA NPs

In order to obtain macrophage membrane vesicles, the extracted membrane was first sonicated for 15 min and then extruded through a membrane filter with a pore size of 400 nm. Then, the macrophage vesicles were mixed with RNA NPs in a 1:1 ratio (membrane protein to polymer ratio) and subjected to ultrasound treatment.

DLS (Malvern Panalytical, England) was used to measure the hydrodynamic size and the zeta potential of MMM vesicles, RNA NPs, and MMM/RNA NPs.

### Quantification of Cytokine Targeted Binding

To test cytokine‐targeting binding capacity, 2 ng mL^−1^ mouse S100A9 was mixed with 0 to 400 µg mL^−1^ of MM/RNA NPs or MMM/RNA NPs in 1×PBS containing 10％FBS. The mixtures were incubated at 37 °C for 1 h. NPs were then removed by centrifugation at 12 000 rpm for 15 min. Cytokine concentration in the supernatant was measured using commercial mouse S100A9 enzyme‐linked immunosorbent assay (ELISA) kit (ELK Biotechnology, Wuhan, China).

### Inflammatory Cytokine Assay in Neutrophils

Neutrophils were inoculated in 24‐well plates at 1 × 10^5^ cells per well and incubated for 12 h. The control and treatment groups were then treated with 100 ng mL^−1^ LPS. The treatment groups were treated with MMM/RNA NPs or MM/RNA NPs at various concentrations for 24 h. Then, the supernatant was taken after centrifugation of the culture solution, and the expression levels of TNF‐α, IL‐6, IL‐1β and MPO in the supernatant were determined by ELISA.

### Cytotoxicity Assay

MNHCs, HUVECs, SMCs, and RAW 264.7 cells were each inoculated in 96‐well plates at a density of 1 × 10^4^ cells per well and incubated for 12 h. Cells were treated with different concentrations of RNA NPs or MMM/RNA NPs for 24 h. Cell viability was measured using the CCK‐8 assay.

### Animals and Treatment

The present study was approved by the Institution Ethics Committee of Anhui Medical University (Anhui, China) (No. LLSC20230846) and performed in accordance with the guidelines from Directive 2010/63/EU of the European Parliament. Male C57 BL/6 mice (21‐25 g) at 7–8 weeks of age were purchased from Jiangsu Jicui Pharmachem Biotechnology Co. C57 BL/6 mice were used to perform the MIRI surgery. After intraperitoneal injection of chloral hydrate in mice, open‐heart surgery was performed. The left anterior descending branch (LAD) was closed with 7‐0 silk sutures for 30 min and relaxed for 24 h. Then, the hearts and blood of mice were collected for the following experiments. A dosage of 2.0 mg kg^−1^ of saline, free siRNA, RNA NPs, MM/RNA NPs, and MMM/RNA NPs was administered via the tail vein.

### qPCR Assay

S100A9 expression assay was performed using qPCR. Table S3 (Supporting Information) lists the primers used in the experiments. RNA was extracted from MNHCs using Trizol reagent (Vazyme Biotech, China). HiScript III qRT SuperMix (Vazyme Biotech, China) was used to synthesize cDNA.

### Cardiac Function Assessment

Mice were anaesthetized with isoflurane, and then M‐mode images were obtained by using a Vevo 2100 477 transthoracic echocardiograph (Visualsoics, Canada). LVEF and LVFS were calculated using the biplane Simpson method and the corresponding software.

### Statistical Analysis

Data are shown as mean ± standard deviation (SD). Student's t‐test was used to assess significant differences between two groups. For comparisons among multiple groups, analysis of variance (ANOVA) was conducted, followed by Tukey's post hoc test for correction. Statistical analyses were performed using GraphPad Prism 8 (GraphPad, La Jolla, CA, USA). All group numbers and detailed significance values were presented in the figure or their legends.

## Conflict of Interest

The authors declare no conflict of interest. The graphical abstract, Figure 1, Figure 4E, Figure 5A, 6A, 7A and Figure S2A were drawn using Figdraw (www.figdraw.com).

## Author Contributions

H.L., J.W., and Z.C. contributed equally to this work, and are the co‐first authors. H.L., J.W., R.L., Y.L., and J.X. designed and completed experiments. H.L., J.W., J.W., Y.J., Z.X., Y.H., and P.S. collected and analyzed the data. H.L. and J.W. wrote the manuscript.

## Supporting information



Supporting Information

## Data Availability

The data that support the findings of this study are available from the corresponding author upon reasonable request.
