# Supplementary material for: Engineered Macrophage Membrane‐Coated S100A9‐siRNA for Ameliorating Myocardial Ischemia‐Reperfusion Injury
Source: Adv Sci (Weinh). 2024 Sep 12;11(41):2403542. doi: 10.1002/advs.202403542 (PMC11538685; doi:10.1002/advs.202403542)
Supplement: Supplementary file 1 — Supporting Information [file ADVS-11-2403542-s001.docx]

Supporting Information

**Engineered Macrophage Membrane-Coated S100A9-siRNA for Ameliorating Myocardial Ischemia-Reperfusion Injury**

*He Lu^#^, Junzhuo Wang^#^, Ziwei Chen^#^, Jing Wang, Yaohui Jiang, Zequn Xia, Ya Hou, Pingping Shang, Rutian Li^*^, Yuyong Liu^*^, Jun Xie^*^*


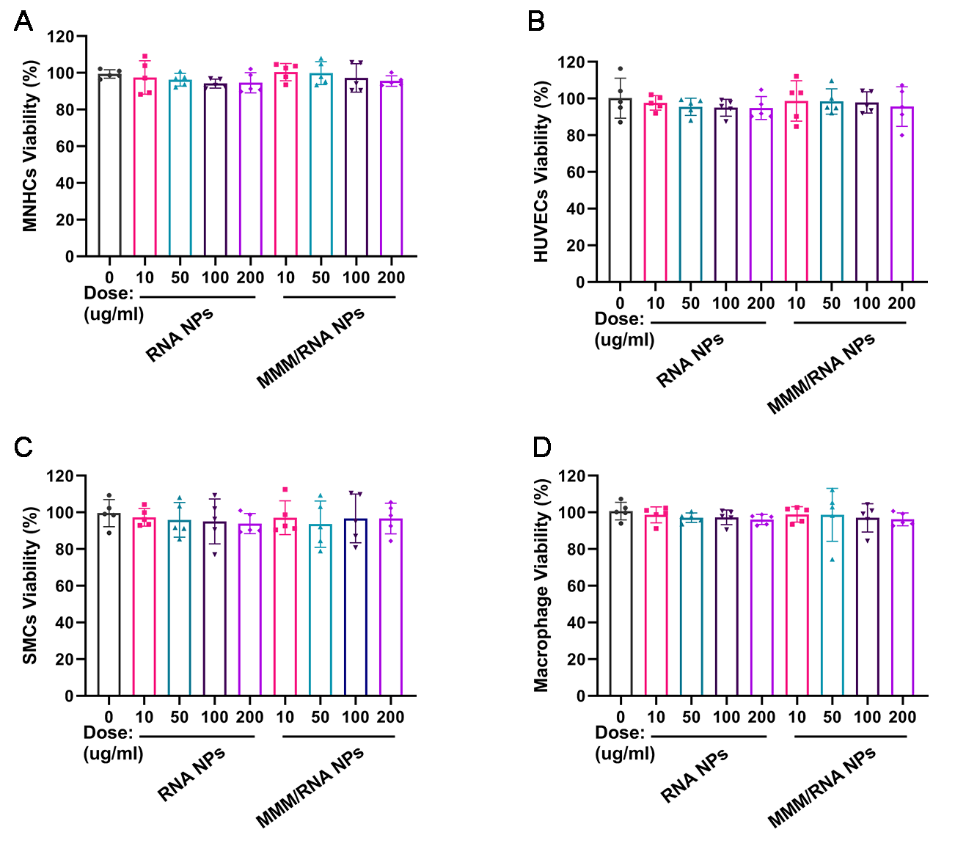


**Figure S1.** CCK-8 detection of cell activity at different concentrations of RNA NPs and MMM/RNA NPs. Cell viability of A) MNHCs, B) HUVECs, C) SMCs and D) RAW 264.7 cells after incubation with various doses of RNA NPs and MMM/RNA NPs for 24 h measured by CCK-8 (mean ± SD, n=5).


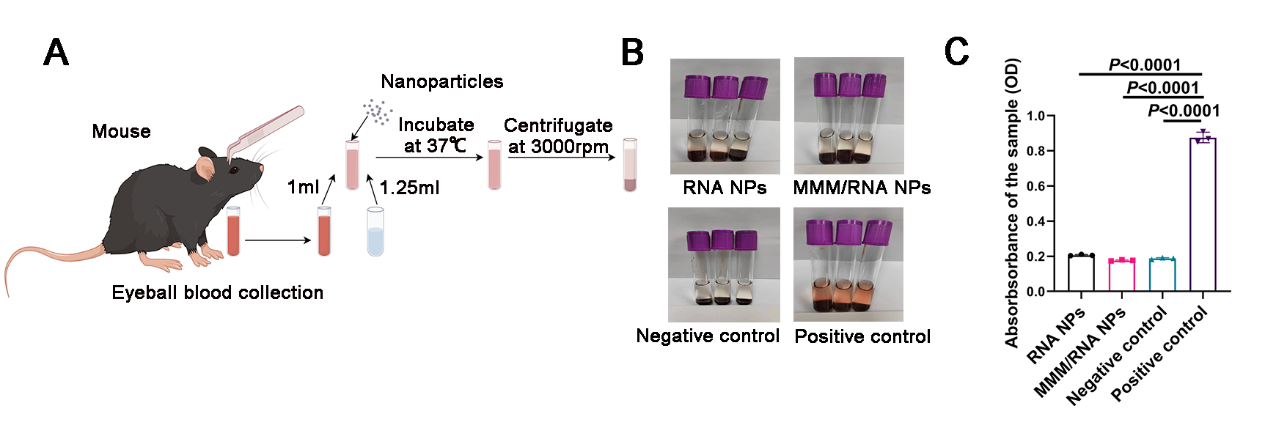


**Figure S2.** A) Schematic illustration of the hemolysis test. B) Centrifuged red blood cells after incubation with RNA NPs and MMM/RNA NPs. C) The absorbance of the blood sample incubated with RNA NPs and MMM/RNA NPs measured at 540 nm (n = 3, mean ± SD). one-way ANOVA, Tukey’s multiple comparisons test.


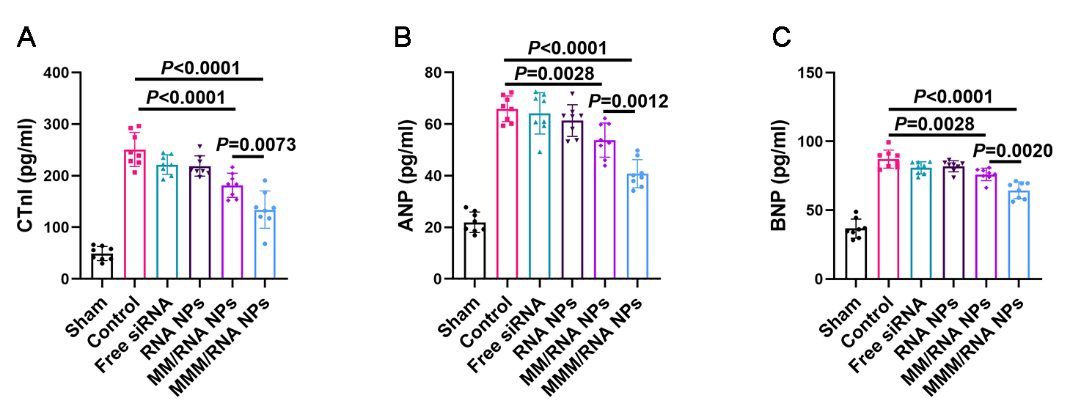


**Figure S3.** MMM/RNA NPs can protect the myocardium. A) Detection of CTnI concentrations in serum using ELISA kits (n=8). B,C) Detection of ANP and BNP concentrations in serum using ELISA kits (n=8). Results are presented as mean ± SD. One-way ANOVA was used for the analysis in A-C).

**Table S1**

Hemagglutinin（HA） gene sequence

| Transcript number | Influenza HA |
| --- | --- |
| Origin | AGCAAAAGCAGGGGATACAAAATGAACACTCAAATCCTGGTTTTCGCCCTTGTGGCAGTCATTCCCACAAATGCAGACAAAATTTGTCTTGGACATCATGCTGTATCAAATGGCACCAAAGTAAACACACTCACTGAGAGAGGAGTAGAAGTTGTCAATGCAACGGAAACAGTGGAGCGGACAAACATCCCCAAAATTTGCTCAAAAGGGAAAAGAACCACTGATCTTGGCCAATGCGGACTGTTAGGGACCATTACCGGACCACCTCAATGCGACCAATTTCTAGAATTTTCAGCTGATCTAATAATCGAGAGACGAGAAGGAAATGATGTTTGTTACCCGGGGAAGTTTGTTAATGAAGAGGCATTGCGACAAATCCTCAGAGGATCAGGTGGGATTGACAAAGAAACAATGGGATTCACATATAGTGGAATAAGGACCAACGGAACAACTAGTGCATGTAGAAGATCAGGGTCTTCATTCTATGCAGAAATGGAGTGGCTCCTGTCAAATACAGACAATGCTTCTTTCCCACAAATGACAAAATCATACAAAAACACAAGGAGAGAATCAGCTCTGATAGTATGGGGAATCCACCATTCAGGATCAACCACCGAACAGACCAAACTATATGGGAGTGGAAATAAACTGATAACAGTCGGGAGTTCCAAATATCATCAATCTTTTGTGCCGAGTCCAGGAACACGACCGCAGATAAATGGCCAGTCCGGACGGATTGATTTTCATTGGTTGATCTTGGATCCCAATGATACAGTTACTTTTAGTTTCAATGGGGCTTTCATAGCTCCAAATCGTGCCAGCTTCTTGAGGGGAAAGTCCATGGGGATCCAGAGCGATGTGCAGGTTGATGCCAATTGCGAAGGGGAATGCTACCACAGTGGAGGGACTATAACAAGCAGATTGCCTTTTCAAAACATCAATAGCAGAGCAGTTGGCAAATGCCCAAGATATGTAAAACAGGAAAGTTTATTATTGGCAACTGGGATGAAGAACGTTCCCGAACCTTCCAAAAAAAGGAAAAAAAGAGGCCTGTTTGGCGCTATAGCAGGGTTTATTGAAAATGGTTGGGAAGGTCTGGTCGACGGGTGGTACGGTTTCAGGCATCAGAATGCACAAGGAGAAGGAACTGCAGCAGACTACAAAAGCACCCAATCGGCAATTGATCAGATAACCGGAAAGTTAAATAGACTCATTGAGAAAACCAACCAGCAATTTGAGCTAATAGATAATGAATTCACTGAGGTGGAAAAGCAGATTGGCAATTTAATTAACTGGACCAAAGACTCCATCACAGAAGTATGGTCTTACAATGCTGAACTTATTGTGGCAATGGAAAACCAGCACACTATTGATTTGGCTGATTCAGAGATGAACAGGCTGTATGAGCGAGTGAGGAAACAATTAAGGGAAAATGCTGAAGAGGATGGCACTGGTTGCTTTGAAATTTTTCATAAATGTGACGATGATTGTATGGCTAGTATAAGGAACAATACTTATGATCACAGCAAATACAGAGAAGAAGCGATGCAAAATAGAATACAAATTGACCCAGTCAAATTGAGTAGTGGCTACAAAGATGTGATACTTTGGTTTAGCTTCGGGGCATCATGCTTTTTGCTTCTTGCCATTGCAATGGGCCTTGTTTTCATATGTGTGAAGAACGGAAACATGCGGTGCACTATTTGTATATAAGTTTGGAAAAACACCCTTGTTTCTACT |

**Table S2**

RAGE gene sequence

| Transcript number | NM_007425.3 |
| --- | --- |
| Origin | GGGAAGAGGGGCAGACAGAACCTGGAGCCTGGGAAGGAAGCACCATGCCAGCGGGGACAGCAGCTAGAGCCTGGGTGCTGGTTCTTGCTCTATGGGGAGCTGTAGCTGGTGGTCAGAACATCACAGCCCGGATTGGAGAGCCACTTGTGCTAAGCTGTAAGGGGGCCCCTAAGAAGCCGCCCCAGCAGCTAGAATGGAAACTGAACACAGGAAGAACTGAAGCTTGGAAGGTCCTCTCTCCCCAGGGAGGCCCCTGGGACAGCGTGGCTCGAATCCTCCCCAATGGTTCCCTCCTCCTTCCAGCCACTGGAATTGTCGATGAGGGGACTTTCCGGTGTCGGGCAACTAACAGGCGAGGGAAGGAGGTCAAGTCCAACTACCGAGTCCGAGTCTACCAGATTCCTGGGAAGCCAGAAATTGTGGATCCTGCCTCTGAACTCACAGCCAGTGTCCCTAATAAGGTGGGGACATGTGTGTCTGAGGGAAGCTACCCTGCAGGGACCCTTAGCTGGCACTTAGATGGGAAACTTCTGATTCCCGATGGCAAAGAAACACTCGTGAAGGAAGAGACCAGGAGACACCCTGAGACGGGACTCTTTACACTGCGGTCAGAGCTGACAGTGATCCCCACCCAAGGAGGAACCCATCCTACCTTCTCCTGCAGTTTCAGCCTGGGCCTTCCCCGGCGCAGACCCCTGAACACAGCCCCCATCCAACTCCGAGTCAGGGAGCCTGGGCCTCCAGAGGGCATTCAGCTGTTGGTTGAGCCTGAAGGTGGAATAGTCGCTCCTGGTGGGACTGTGACCTTGACCTGTGCCATCTCTGCCCAGCCCCCTCCTCAGGTCCACTGGATAAAGGATGGTGCACCCTTGCCCCTGGCTCCCAGCCCTGTGCTGCTCCTCCCTGAGGTGGGGCACGAGGATGAGGGCACCTATAGCTGCGTGGCCACCCACCCTAGCCACGGACCTCAGGAAAGCCCTCCTGTCAGCATCAGGGTCACAGAAACCGGCGATGAGGGGCCAGCTGAAGGCTCTGTGGGTGAGTCTGGGCTGGGTACGCTAGCCCTGGCCTTGGGGATCCTGGGAGGCCTGGGAGTAGTAGCCCTGCTCGTCGGGGCTATCCTGTGGCGAAAACGACAACCCAGGCGTGAGGAGAGGAAGGCCCCGGAAAGCCAGGAGGATGAGGAGGAACGTGCAGAGCTGAATCAGTCAGAGGAAGCGGAGATGCCAGAGAATGGTGCCGGGGGACCGTAAGAGCACCCAGATCGAGCCTGTGTGATGGCCCTAGAGCAGCTCCCCCACATTCCATCCCAATTCCTCCTTGAGGCACTTCCTTCTCCAACCAGAGCCCACATGATCCATGCTGAGTAAACATTTGACACGGTGTG |

**Table S3**

Primers used for qPCR

| Name | Primers |  |
| --- | --- | --- |
| Mouse S100A9 | Forward  Reverse | TGGGCTTACACTGCTCTTACC  GGTTATGCTGCGCTCCATCT |
